# Living well with dementia: a systematic review and correlational meta-analysis of factors associated with quality of life, well-being and life satisfaction in people with dementia – CORRIGENDUM

**DOI:** 10.1017/S0033291720001713

**Published:** 2021-07

**Authors:** Anthony Martyr, Sharon M. Nelis, Catherine Quinn, Yu-Tzu Wu, Ruth A. Lamont, Catherine Henderson, Rachel Clarke, John V. Hindle, Jeanette M. Thom, Ian Rees Jones, Robin G. Morris, Jennifer M. Rusted, Christina R. Victor, Linda Clare

**Affiliations:** 1 School of Psychology, University of Exeter, EX4 4QG, UK; 2 PenCLAHRC, Institute of Health Research, University of Exeter Medical School, EX1 2LU, UK; 3 Personal Social Services Research Unit, London School of Economics and Political Science, WC2A 2AE, UK; 4 School of Psychology, University of Sussex, BN1 9RH, UK; 5 School of Education and Social Work, University of Sussex, BN1 9RH, UK; 6 School of Psychology, Bangor University, LL57 2AS, UK; 7 Department of Care for the Elderly, Betsi Cadwaladr University Health Board, Llandudno, LL30 1LB, UK; 8 School of Medical Sciences, University of New South Wales, NSW 2052, Australia; 9 Wales Institute of Social and Economic Research, Data and Methods, Cardiff University, CF10 3BB, UK; 10 Department of Psychology, King's College London Institute of Psychiatry, Psychology and Neuroscience, London, SE5 8AF, UK; 11 Department of Clinical Sciences, Brunel University, UB8 3PH, UK; 12 Wellcome Centre for Cultures and Environments of Health, University of Exeter, Exeter, UK

Two errors have been identified in the analysis reported in the above article.

Figure 1: The number of studies included in quantitative synthesis (meta-analysis) was 198 rather than 199.

Figure 2: The authors reanalysed data for “Presence of religious beliefs/spirituality” as it was noticed that three data points were entered into the meta-analysis software with incorrect direction. The meta-analysis effect size originally reported was *r* = 0.35 (95% CI 0.12, 0.55), *p* = .0035, *I*^2^ = 88.453, (*I*^2^ was included in Supplementary Table 13a only). Reanalysis indicated a correct effect size of: *r* = 0.15 (−0.12, 0.15), *p* = .8280, *I*^2^ = 62.101. Therefore, presence of religious beliefs/spirituality had a negligible association with better quality of life, rather than a moderate association as originally stated (p.2134).

The authors apologise for these errors.

## References

[ref1] Martyr, A., Nelis, S. M., Quinn, C., Wu, Y-T., Lamont, R. A., Henderson, C., Clarke, R., Hindle, J. V., Thom, J. M., Jones, I. R., Morris, R. G., Rusted, J. M., Victor, C. R., and Clare, L. (2018). Living well with dementia: a systematic review and correlational meta-analysis of factors associated with quality of life, well-being and life satisfaction in people with dementia. Psychological Medicine, 48 (13), 2130-2139. doi: 10.1017/S0033291718000405 29734962

